# A novel LncRNA SPIRE1/miR-181a-5p/PRLR axis in mandibular bone marrow–derived mesenchymal stem cells regulates the Th17/Treg immune balance through the JAK/STAT3 pathway in periodontitis

**DOI:** 10.18632/aging.204895

**Published:** 2023-07-24

**Authors:** Bingyi Shao, Duo Zhou, Jie Wang, Deqin Yang, Jing Gao

**Affiliations:** 1 Northern Department of Endodontics, Stomatological Hospital of Chongqing Medical University, Chongqing 401147, China; 2 Chongqing Key Laboratory of Oral Diseases and Biomedical Sciences, Chongqing 401147, China; 3 Chongqing Municipal Key Laboratory of Oral Biomedical Engineering of Higher Education, Chongqing 401147, China

**Keywords:** periodontitis, LncRNA SPIRE1, miR-181a-5p, PRLR, mesenchymal stem cells, immunomodulation, STAT3 pathway

## Abstract

Periodontitis is a microbial-related chronic inflammatory disease associated with imbalanced differentiation of Th17 cells and Treg cells. Bone marrow-derived mesenchymal stem cells (BM-MSCs) possess wide immunoregulatory properties. Long noncoding RNAs (lncRNAs) and microRNAs (miRNAs) contribute to the immunomodulation in the pathological mechanisms of inflammatory diseases. However, critical lncRNAs/miRNAs involved in immunomodulation of mandibular BM-MSCs largely remain to be identified. Here, we explored the molecular mechanisms behind the defective immunomodulatory ability of mandibular BM-MSCs under the periodontitis settings. We found that mandibular BM-MSCs from *P. gingivalis*-induced periodontitis mice had significantly reduced expression of LncRNA SPIRE1 than that from normal control mice. LncRNA SPIRE1 knockdown in normal BM-MSCs caused Th17/Treg cell differentiation imbalance during the coculturing of BM-MSCs and CD4 T cells. In addition, LncRNA SPIRE1 was identified as a competitive endogenous RNA that sponges miR-181a-5p in BM-MSCs. Moreover, miR-181a-5p inhibition attenuated the impact of LncRNA SPIRE1 knockdown on the ability of BM-MSCs in modulating Th17/Treg balance. Prolactin receptor (PRLR) was validated as a downstream target of miR-181a-5p. Notably, targeted knockdown of LncRNA SPIRE1 or PRLR or transfection of miR-181a-5p mimics activated the JAK/STAT3 signaling in normal BM-MSCs, while treatment with STAT3 inhibitor C188-9 restored the immunomodulatory properties of periodontitis-associated BM-MSCs. Furthermore, BM-MSCs with miR-181a-5p inhibition or PRLR-overexpression showed enhanced *in vivo* immunosuppressive properties in the periodontitis mouse model. Our results indicate that the JAK/STAT3 pathway is involved in the immunoregulation of BM-MSCs, and provide critical insights into the development of novel targeted therapies against periodontitis.

## INTRODUCTION

Periodontitis is a severe gum infection that can lead to tooth loss and other serious health complications, such as diabetes, rheumatoid arthritis, and infective endocarditis [1, 2]. As a multifactorial inflammatory disease, periodontitis affects gingiva and deeper tissues like bone and periodontal ligaments [3]. A localized inflammation of the gingiva, induced by the microorganisms of the dental plaque, such as *Porphyromonas gingivalis*, can progress to the resorption of periodontal tissues and further loss of tissue structure and function [4, 5]. The bacteria infection initiated periodontal diseases were reported to correlate with the interaction of pathogens, host inflammation, and immune responses [6, 7]. T-helper 17 (Th17) cells have been identified as a unique CD4^+^ T-helper subset, characterized by IL-17 production that promotes tissue inflammation [8, 9]. Regulatory T (Treg) cells are a specialized lineage of suppressive CD4 T cells that act as critical negative regulators of inflammation in various biological contexts [10]. Treg cells exposed to inflammatory conditions acquire strongly enhanced suppressive function [10]. The imbalance of Th17/Treg has been identified in patients with chronic periodontitis, which was demonstrated by upregulation of Th17 and downregulation of Treg cells through immune-mediated tissue destruction [11]. It was reported that Th17 cells play a destructive role in the immune balance of periodontitis, whereas *Porphyromonas gingivalis* infection could be associated with Tregs dysregulation in atherosclerosis [12, 13]. Therefore, it is paramount to understand the molecular mechanisms underlying the pathogenesis of Th17/Treg imbalance in the development and treatment of periodontitis.

Mesenchymal stem cells (MSCs) are multipotent stem cells characterized by self-renewal, production of clonal cell populations, and multilineage differentiation [14]. They exist in nearly all tissues and play a significant role in tissue repair and regeneration. In addition, through interaction with various immune cells, MSCs possess extensive immunosuppressive properties, and their immunosuppressive properties from different tissues are being explored for promising therapeutic application in immune disorders [15, 16]. For example, the mandibular gingiva-derived mesenchymal stem cells (GMSCs), isolated from easily accessible tissues of oral mucosa, possess self-renewal, multilineage differentiation, and immunomodulatory properties [17, 18]. Systemic administration of GMSCs has been proven to promote skin wound healing and mitigate chemotherapy-induced oral mucositis, as well as attenuating experimental colitis in the mouse model [18]. GMSCs also promoted Treg cell polarization and inhibited Th17 cell polarization [19, 20]. Possessing the excellent self-renewal ability and multi-lineage differentiation potential, mandibular bone marrow-derived mesenchymal stem cells (BM-MSCs) are another source of MSCs that have a high osteogenic ability [21, 22]. They are considered one of the most suitable multipotent stem cells for bone regeneration; therefore, they are widely used for comparisons of efficiency with other cell sources [21, 22]. Periodontal wound healing by transplantation of mandibular BM-MSCs was reported to be applicable in conjugation with the chitosan/anorganic bovine bone (C/ABB) scaffold to support the healing/repair of one-wall critical size periodontal defects in beagles [23]. However, the potential contributions of mandibular BM-MSCs in periodontal repair in terms of immunomodulation have not been intensively investigated.

Noncoding RNAs (ncRNAs) are a class of RNAs that do not code for proteins, but they were identified to play important roles in the differentiation and function of MSCs [24]. Two of the most important ncRNAs currently identified in odontogenic differentiation are microRNAs (miRNAs) and long noncoding RNAs (lncRNAs). miRNAs mainly regulate gene expression through interacting with the 3′untranslated region (UTR) of certain transcript. Whereas, lncRNAs inhibit the negative effect of miRNAs, thereby inhibiting their negative effect on gene expression [25]. The fundamental impacts of miRNAs and lncRNAs in differentiation of stem cells both in normal and diseased conditions have been revealed [25], while the direct roles of miRNAs/lncRNAs in immunomodulation in the pathological mechanisms of autoimmune-related diseases were also shown [26]. For example, silencing of the LncRNA NEAT1 was reported to improve the Treg/Th17 imbalance in preeclampsia via the miR-485-5p/AIM2 axis [27]. With the help of high-throughput sequencing and microarrays, increasing evidence has confirmed the roles of lncRNAs as master regulators of various biological processes [28], including the development of periodontitis [29–31]. Nevertheless, critical lncRNAs in mandibular BM-MSCs largely remain to be identified, and their associations to immunomodulation require more intensive studies.

The current study was conducted to explore the possible roles of SPIRE1, a novel lncRNA, in regulating the immunomodulation ability of mandibular BM-MSCs on the balance of Th17/Treg *in vitro* and *in vivo*. Our further investigation showed that LncRNA SPIRE1 is a competitive endogenous RNA (ceRNA) that sponges miR-181-5p, which targeted prolactin receptor (PRLR) to regulate the immunosuppressive functions of BM-MSCs through the Janus kinase (JAK)/signal transducer and activator of transcription 3 (STAT3) pathway. These underlying molecular mechanisms can aid the development of BM-MSCs-based therapeutics for periodontitis therapies.

## MATERIALS AND METHODS

### Animals

Female C57BL/6J mice (6–8 weeks old, *n* = 200) were purchased from Beijing Vital River Laboratory Animal Technology Co. Ltd. (Beijing, China) and kept in specific-pathogen-free (SPF) conditions in the Laboratory Animal Center of Chongqing Medical University. Mice were housed in the room with a 12-hour alternating light-dark cycle and were fed sufficient diet and water *ad libitum* throughout the experimental period. All experimental protocols were performed in accordance with the Guidelines for Care and Use of Laboratory Animals of Chongqing Medical University and approved by the Animal Ethics Committee of the Chongqing Medical University (approved protocol number 2020-067). Humane endpoints were used during the animal experiment assays; if any mice had lost 20% of their body weight before the end of experiment, they were thus immediately euthanized. Mice were sacrificed after injection of sodium pentobarbital (100 mg/kg body weight).

### MSC isolation and culture

Isolation of MSCs from mouse jaw (mandibular) was performed as previously described [32]. Briefly, mice were euthanized with sodium pentobarbital and the mandibular bones were separated. The nucleated cells from mandibular bones were obtained by digestion with 3 mg/mL collagenase type I (Sigma-Aldrich; St. Louis, MO, USA) and 4 mg/mL dispase II (Sigma-Aldrich) for 60 min at 37°C. After passing the 70 μm cell strainers (BD Bioscience, San Jose, CA, USA), single-cell suspensions were cultured in 35 mm culture dishes filled with Dulbecco’s Modified Eagle Medium (DMEM; Thermo Fisher Scientific, Waltham, MA, USA), which was supplemented with 10% fetal bovine serum (FBS; Sigma), 1% penicillin and streptomycin (Accuref Scientific, Xi’an, China), and 1× GlutaMax^®^ (Thermo Fisher Scientific). Dishes were placed in 37°C incubators with 5% CO_2_, and the culture medium was replaced every 3 days. The initial adherent single colonies were considered as passage 0 (P0) MSCs, and were collected to be passed to other passages for subsequent experiments.

### Manipulation of gene expression in MSCs

Mandibular BM-MSCs were transfected with the short hairpin RNA (shRNA), miRNA mimics or inhibitors, and mammalian gene over-expressing plasmids as indicated, using the Lipofectamine 3000 Transfection Reagent (Thermo Fisher Scientific) following the manufacturer’s instructions. The DNA coding sequence for LncRNA SPIRE1 was cloned into the pcDNA^™^3.1 (+) Mammalian Expression Vector (Thermo Fisher Scientific). For shRNAs design, sequences of SPIRE1 and PRLP were obtained from the National Center for Biotechnology Information (NCBI) and shRNA sequences were designed using the online tool provided by the Life Technologies (https://rnaidesigner.thermofisher.com/rnaiexpress/sort.do). The shRNA sequences for PRLR (GCCACCTAC CATAACTGATGT) and SPIRE1 (GGTGTTTGACA GCATGTTA) were cloned into lentiviral vector. The empty vector and the vector expressing shRNA-NC were used as negative controls. The miR-181a-5p mimic (AACAUUCAACGCUGUCGGUGAGU) and inhibitor (ACUCACCGACAGCGUUGAAUGUU), as well as the corresponding negative controls (NC-mimic, #miR1N0000001-1-10; NC-inhibitor, miR2N0000001-1-10) were chemically synthesized by Guangzhou RiboBio., Ltd (Guangzhou, China). For lentivirus infection of BM-MSCs, cells at the logarithmic growth phase were infected by lentivirus with a Multiplicity of Infection (MOI) of 10. The lentiviral vector pcDNA3.0 (Invitrogen; Carlsbad, CA, USA) was used for lentivirus generation in Lenti-X^™^ 293T cells (Takara Bio Inc., Kusatsu, Gunma Prefecture, Japan). The vector constructions, verification by sequencing, virus packaging and collection of the corresponding viral supernatants were performed by Hanyin Biotechnology Limited Company (Shanghai, China). At 48 hours after transfection or infection, BM-MSCs with confirmed gene manipulation were used for further experiments.

### T cells isolation and co-culture with MSCs

Mouse splenic CD4^+^ T lymphocytes were isolated using a mouse CD4^+^ T Cell Isolation Kit (Miltenyi Biotec; Auburn, CA, USA) with magnetic beads following the manufacturer’s instructions. Mandibular BM-MSCs at passage 2–4 from control mice or periodontitis mice after the indicated manipulation of gene expression were seeded on 24-well plates at a concentration of 1 × 10^5^ cells/well and incubated overnight before they were co-cultured with activated mouse CD4^+^ T cells. Mouse T lymphocytes were pre-activated in RPMI 1640 medium supplemented with 10% heat-inactivated FBS, 50 μM 2-mercaptoethanol, 10 mM HEPES, 2 mM L-glutamine, 100 U/mL penicillin, and 100 mg/mL streptomycin (all reagents were from Thermo Fisher Scientific) by plate-bound anti-CD3 antibody (1:1000 dilution; #ab16669; Abcam, USA) and soluble anti-CD28 antibody (1:5000 dilution; #ab283860, Abcam, USA) for 2–3 days in 24-well plates. Recombinant human TGF-β1 (2 ng/mL; R&D Systems, Minneapolis, MN, USA) and IL-2 (2 ng/mL; R&D Systems) were added into the culture medium to induce Treg differentiation; while recombinant human TGF-β1 (2 ng/mL; R&D Systems) and IL-6 (50 ng/mL; R&D Systems) were added to induce Th17 differentiation. The activated mouse CD4^+^ T cells were loaded on the adherent BM-MSCs and co-cultured for further 2–3 days. The supernatant and T cells on top layers were collected for further analyses.

### Enzyme-linked immunosorbent assay (ELISA)

Mouse blood samples were collected from orbital sinus into 1.5 mL centrifuge tubes after anesthesia of mice with 2% isoflurane (0.41 mL/min at 4 L/min fresh gas flow). Successful induction of anesthesia was confirmed when mice were fully slack and had no response to pain stimuli, but presented normal breath and beats. After blood samples collection (>0.5 ml), mice were sacrificed for tissue samples collection. Plasma samples were separated by centrifugation at 12000 g for 10 min at 4°C and frozen at −80°C until use. The levels of IL-17 (#PI545) and IL-10 (PI522) in plasma and supernatant samples were measured by ELISA using kits from Beyotime, Shanghai, China. All assays were performed following the manufacturer’s instructions.

### Quantitative PCR

Total RNA was extracted from blood samples and T cells using Trizol reagent (Accuref Scientific, Xi’an, China) following the manufacturer’s instructions. The synthesis of cDNA was conducted by the PrimeScript^®^ RT Master Mix Perfect Real Time Reagent Kit (Accuref Scientific, Xi’an, China). Quantitative reverse transcription PCR (qRT-PCR) was performed using a standard protocol from the SYBR Green PCR kit (Accuref Scientific, Xi’an, China) on an AB7500 RT-PCR instrument (Applied Biosystems, Foster City, CA, USA). The sequences of PCR primers for the indicated genes are listed in Supplementary Table 1. The qPCR experiments were conducted with each sample run in triplicates. PCR product specificity was confirmed by melting curve analysis. Gene expression levels were calculated with the 2^−ΔΔCT^ method. MiRNA expression was quantified using the TaqMan MicroRNA Expression Assay (Applied Biosystems, Foster City, CA, USA) per the manufacturer’s instructions. The expression level of miR-181a-5p was normalized to that of the U6 and mRNA was normalized to the GAPDH.

### Luciferase reporter assay

DNA sequences coding the indicated wild type or mutated LncRNA SPIRE1 were cloned into a luciferase-expressing vector pGL3.Basic (Promega, Madison, WI, USA). The miR-181a-5p targeted candidate genes and the binding sites of PRLR were predicted using the online tools STarMirDB and miRWalk (http://mirwalk.umm.uni-heidelberg.de/). The predicted binding sites within the 3′UTR of PRLR mRNA and the corresponding mutated binding region were cloned into the vector pGL3.Basic. These vectors together with the miR-181a-5p mimic or NC mimic were co-transfected into BM-MSCs. At 48 hours after transfection, cells were harvested and subjected to luciferase activity analysis using a Dual-Luciferase Reporter Assay System (Promega, Madison, WI, USA) following the manufacturer’s instructions. The relative luciferase activity was normalized to the luciferase activity of cells transfected with wild type PRLR 3′UTR and NC-mimic.

### Western blot assay

Total proteins were extracted after cell lysis with the radioimmunoprecipitation assay lysis buffer (RIPA; Accuref Scientific, Xi’an, China). A total of 20 μg samples were separated by 10% sodium dodecyl sulfate polyacrylamide gel electrophoresis (SDS-PAGE), transferred to polyvinylidene difluoride (PVDF) membranes. After blocking with tris-buffered saline (TBS) supplemented with 5% nonfat dry milk for 1 hour, the membrane was incubated with the indicated primary antibodies (anti-PRLR, 1:1000 dilution, #ab170935; anti-JAK2, 1:5000 dilution, #ab108596; anti-pSTAT3, 1:1000 dilution, #AB267373; anti-β-actin, 1 μg/ml, #ab8226; all purchased from Abcam) at 4°C overnight. After binding with the secondary horseradish peroxidase (HRP)-conjugated anti-rabbit IgG (1:10,000; #ab6721, Abcam) or anti-mouse IgG (1:10,000; #ab6728, Abcam) for 1 hour at room temperature, the membranes were processed with the enhanced chemiluminescence kit (Accuref Scientific, Xi’an, China). The expressions of targeted proteins were quantified by densitometry using the ImageJ software (National Institutes of Health, Bethesda, MD, USA).

### Flow cytometry

The ratios of CD4^+^CD25^+^FoxP3^+^ Treg cells or CD4^+^IL-17^+^ Th17 cells among total CD4^+^ T cells were determined by flow cytometry following the manufacturer’s protocol. For staining of Treg cells, the collected mouse T cells were stained with FITC-conjugated anti-mouse CD4 antibody (Ab). After surface staining, T cells were fixed and permeabilized using buffer from BD Biosciences, and further stained with PE-conjugated anti-mouse FoxP3 Ab. For staining of Th17 cells, T cells were stimulated with 5 ng/mL Phorbol 12-myristate 13-acetate (PMA) and 500 ng/mL ionomycin for 4 hours in the presence of a protein-transport inhibitor (Golgi Stop; BD Bioscience). Cells were then incubated with FITC-conjugated anti-mouse CD4 Ab. After fixation and permeabilization, cells were stained with PE-conjugated anti-mouse IL-17 Ab. All Abs and other reagents used for flow cytometry were purchased from BioLegend (San Diego, CA, USA).

### Animal models

Periodontitis in mice was induced by inoculation of the well-recognized periodontal pathogen *Porphyromonas gingivalis*, as previously described [33]. The mice were injected with *P. gingivalis* bacterial lipopolysaccharides (LPS; InvivoGen, San Diego, CA, US) into the palatal gingival sulcus of maxillary first molars twice a week for 4 weeks at a dose of 0.5 mg/kg body weight. After the onset of the disease (5–7 weeks after first LPS injection), mandibular BM-MSCs with the indicated manipulation of gene expressions, including transfection of NC inhibitor or miR-181a-5p inhibitor and infection of empty lentivirus vector or PRLR-expressing lentivirus, as well as the mandibular BM-MSCs treated with the STAT3 inhibitor C188-9 (Selleck Chemicals; 20 μM for 48 hours) or vehicle (DMSO), were infused (1 × 10^6^ cells) into periodontitis mice (*n* = 5 for each group) intravenously once per week for 3 weeks. At 2 weeks after the completion of treatments, mice were euthanized using CO_2_ exposure at a rate of 30% of the chamber volume per minute and verified by the heartbeat or breathing arrest followed by 15 min CO_2_ exposure. After mice were sacrificed, the periodontal tissue and mandibular bones were collected for further analyses. The acute colitis mouse model was induced by administering 3% (w/v) dextran sulfate sodium through drinking water, and the modeling process and evaluation of disease activity index followed previous reports [20, 34].

### Histopathological evaluation

Mandibular samples containing the first molars were dissected, fixed in 4% paraformaldehyde, and decalcified in 10% formic acid at room temperature. Some 4 μm serial sections were stained with hematoxylin and eosin (HE) and Masson’s trichrome (Accuref Scientific, Xi’an, China) following the standard protocols. To identify osteoclasts, tartrate-resistant acid phosphatase (TRAP) activity was detected using the TRAP/ALP Kit (Accuref Scientific, Xi’an, China) according to the manufacturer’s instructions.

For multi-differential potential identification of BM-MSCs, cells were cultured in osteogenic (0.01 μM hexadecadrol, 5 mM sodium β-glycerophosphate, 50 mg/L vitamin C) or adipogenic (0.5 μM hexadecadrol, 60 μM indomethacin, 0.5 mM isobutyl methylxanthine, 10 mg/L bovine insulin) medium for 28 days with a medium exchange frequency of once every 3 days. After washing with PBS three times and fixation with 4% paraformaldehyde, osteogenic cultures were stained with Alizarin Red (Accuref Scientific, Xi’an, China), while adipogenic cultures were stained with Oil Red O (Accuref Scientific, Xi’an, China). Histological observations were conducted from photographs captured under an optical microscope (Nikon Instruments, Tokyo, Japan).

### *In vivo* imaging analysis

Mouse maxilla was scanned using micro-computed tomography (micro-CT; SkyScan1174v2; Bruker-μCT, Billerica, MA, USA) with an accuracy of 18 μm before and after the treatments. For visualization, samples were digitally reconstructed. The periodontal bone loss rate, bone volume/total volume (BV/TV), bone surface/volume ratio (BS/BV), and bone mineral density (BMD), bone growth rate were analyzed by the CT Analyser software (Version: 1.17.7.2, Bruker Micro-CT).

### Statistical analysis

All quantitative results are expressed as mean ± standard deviation (SD) with 3–10 replicates per group as indicated in the figure legend. Groups were compared using unpaired, One-way ANOVA followed by Tukey’s multiple comparison. Mann-Whitney test was used to determine statistically significant changes in variables that were not normally distributed. Statistical analyses were performed using SPSS version 19.0 statistical software (IBM Corporation; Armonk, NY, USA). In all cases, *P* < 0.05 was considered statistically significant.

### Availability of data and materials

The datasets used and/or analyzed during the present study are available from the corresponding author upon reasonable request.

## RESULTS

### Periodontitis in mice was associated with downregulated expression of LncRNA SPIRE1

**Figure 1:**
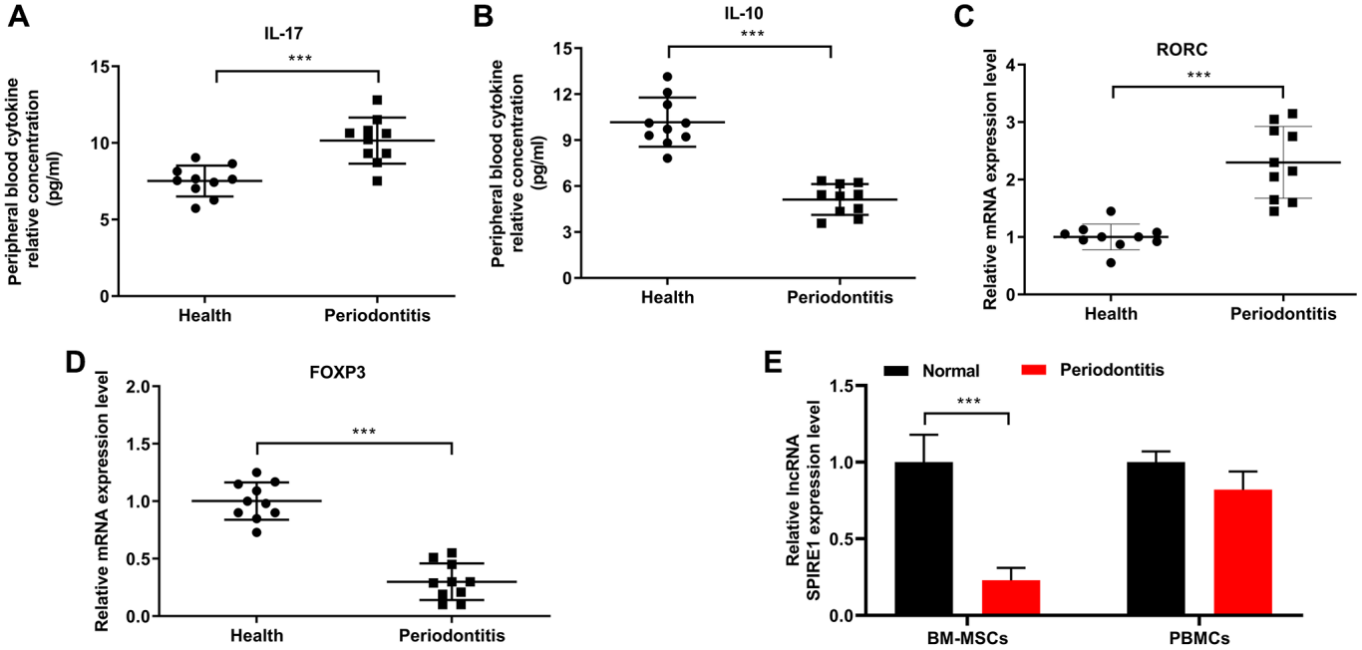
**Chronic periodontitis caused the whole body and local Th17/Treg imbalance, and was associated with downregulated lncRNA SPIRE1 expression in mandibular bone marrow–derived mesenchymal stem cells (BM-MSCs).** (**A**, **B**) The levels of soluble IL-17 (**A**) and IL-10 (**B**) in peripheral blood collected from healthy normal mice or periodontitis mice were measured by ELISA. (**C**, **D**) The expression levels of RORC mRNA (**C**) and FoxP3 mRNA (**D**) in peripheral blood from normal healthy mice or mice with periodontitis were measured by RT-qPCR. (**E**) Mandibular-BM-MSCs (but not blood cells) from periodontitis displayed a lower expression level of LncRNA SPIRE1 than that from normal control. The expression level of LncRNA SPIRE1 in mandibular BM-MSCs and peripheral blood of the indicated groups were quantitated by qPCR. PBMCs, peripheral blood mononuclear cells. *n* = 10 for normal healthy mice; *n* = 10 for periodontitis mice; ^***^P < 0.001, between the indicated groups.

We first examined the immune status associated with periodontitis by comparing the levels of IL-17 and IL-10 from serum samples of healthy normal mice and periodontitis mice. As shown in Figure 1A and 1B, the serum level of IL-17 from the periodontitis group was significantly up-regulated, in comparison to the control healthy group; while that of IL-10 was considerably lower. Consistent with these observation on the imbalanced Th17/Treg immune status of whole body, the peripheral blood demonstrated a significantly more expression of RORC mRNA (Figure 1C) and markedly down-regulated expression of FoxP3 mRNA (Figure 1D) in periodontitis mice. To explore the potential roles of mesenchymal stem cells on the possible local Th17/Treg imbalance, we isolated mandibular BM-MSCs from these mice and confirmed their multi-potential differentiation capacities (Supplementary Figure 1). Notably, the mandibular BM-MSCs from the periodontitis group showed significantly reduced expression of LncRNA SPIRE1, compared with that from the healthy group; whereas peripheral blood samples had largely comparable levels of LncRNA SPIRE1 expression between two groups (Figure 1E), indicating that the local Th17/Treg imbalance might be more severe than that of the whole body.

### Downregulated expression of LncRNA SPIRE1 in mandibular BM-MSCs caused Th17/Treg imbalance

**Figure 2:**
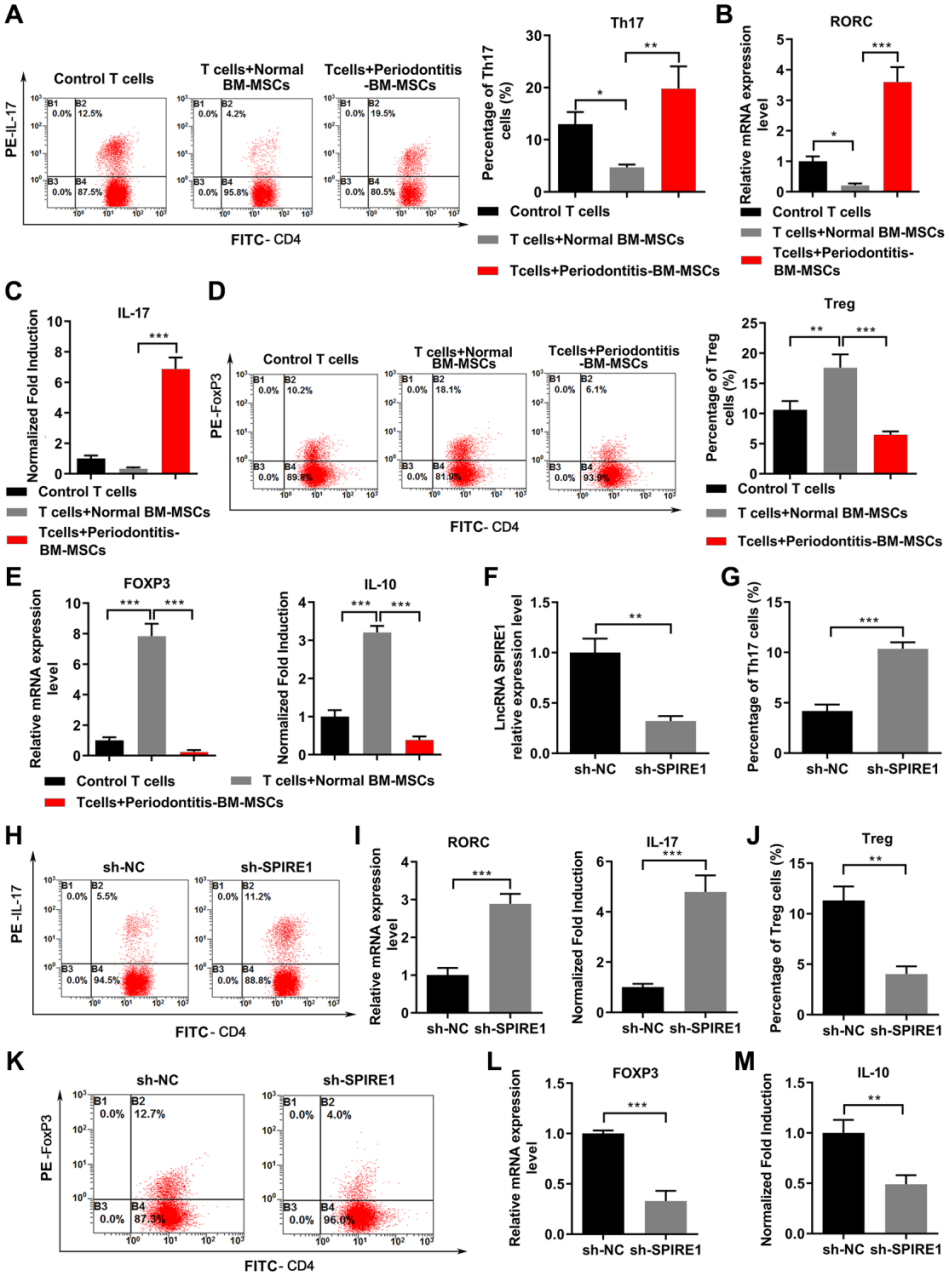
**LncRNA SPIRE1 knockdown impaired the immunomodulatory properties of normal mandibular BM-MSCs by causing Th17/Treg imbalance.** (**A**–**F**) Pre-activated CD4^+^ T cells were cultured alone, or co-cultured with mandibular BM-MSCs from control mice or periodontitis mice under the specific Th17 (**A**–**C**) or Treg (**D**–**E**) polarization condition. Representative flow cytometry profiles of T cells for Th17 (**A**) or Treg (**D**) populations identified as CD4^+^IL-17^+^ cells or CD4^+^FoxP3^+^ cells, respectively, are shown, and their percentages among total CD4^+^ cells were summarized. The mRNA levels of RORC (**B**) or FoxP3 (**E**) in total T cells and the soluble levels of IL-17 (**C**) or IL-10 (**E**) in co-cultured supernatants were quantitated by qPCR or ELISA, respectively. (**F**) The silence-efficacy of SPIRE1 in normal mandibular BM-MSCs was confirmed by qPCR analysis of cells transfected with negative control (NC)-shRNA or LncRNA SPIRE1-specific shRNA. (**G**–**M**) NC-shRNA or SPIRE1-shRNA transfected normal mandibular BM-MSCs were co-cultured with the pre-activated CD4^+^ T cells under the Th17 (**G**–**I**) or Treg (**J**–**M**) polarization condition. Representative flow cytometry profiles of Th17 (**H**) and Treg (**K**) populations, and their percentages are shown. The mRNA levels of RORC (**I**) or FoxP3 (**L**), and the levels of IL-17 (I) or IL-10 (**M**) in supernatants were quantitated by qPCR and ELISA, respectively. *n* = 3 for each group; ^**^P < 0.01; ^***^P < 0.001, between the indicated groups.

To evaluate the impact of periodontitis on the immunomodulation ability of BM-MSCs, we co-cultured the mandibular BM-MSCs obtained from the healthy normal mice or periodontitis mice with mouse activated splenic CD4^+^ T cells, and measured their differentiation toward Th17 cells or Treg cells under the respective polarization condition. Compared with the control T cells without co-culturing with BM-MSCs, the T cells co-cultured with normal BM-MSCs markedly reduced the percentage of CD4^+^IL-17^+^ Th17 population (Figure 2A), which was accompanied with reduced expression of Th17-specific transcription factor RORC (Figure 2B) and reduced production of soluble IL-17 (Figure 2C). On the contrary, the T cells co-cultured with periodontitis BM-MSCs had significantly increased ratio of Th17 population (Figure 2A), profoundly upregulated expression of RORC mRNA (Figure 2B) and secretion of IL-17 (Figure 2C). Under the Treg polarization condition, the periodontitis BM-MSCs behaved dramatically different from the normal BM-MSCs, as the observations on higher percentage of CD4^+^CD25^+^FoxP3^+^ Treg population (Figure 2D), as well as the higher expression of FoxP3 mRNA and IL-10 production in culture medium (Figure 2E), in comparison to the group with the T cells co-cultured with normal BM-MSCs. The FoxP3 mRNA and soluble IL-10 levels from the group with the T cells co-cultured with periodontitis BM-MSCs were drastically diminished to the extents even lower than the control T cells without BM-MSC co-culturing.

To further evaluate the contribution of LncRNA SPIRE1 knockdown on the immunomodulation ability of BM-MSCs, we tested the immunomodulation ability of normal mandibular BM-MSCs after shRNA-mediated LncRNA SPIRE1 knockdown (Figure 2F). As expected, silence of LncRNA SPIRE1 in BM-MSCs rendered a higher percentage of Th17 cells (Figure 2G and Figure 2H), a higher level of RORC mRNA, more production of IL-17 (Figure 2I) in CD4^+^ T cells under the Th17 polarization condition, and a lower percentage of Tregs (Figure 2J and Figure 2K), reduced expression of FoxP3 mRNA (Figure 2L) and secretion of IL-10 (Figure 2M) in CD4^+^ T cells under the Treg polarization condition. Taken together, these results suggested that LncRNA SPIRE1 knockdown in mandibular BM-MSCs from normal controls enhanced Th17 cell function and suppressed Treg cell function during MSCs-T cells coculturing.

### LncRNA SPIRE1 controlled the expression level of miR-181a-5p, whose suppression rescued the ability of LncRNA SPIREI-knockdown BM-MSCs in immune modulation

**Figure 3:**
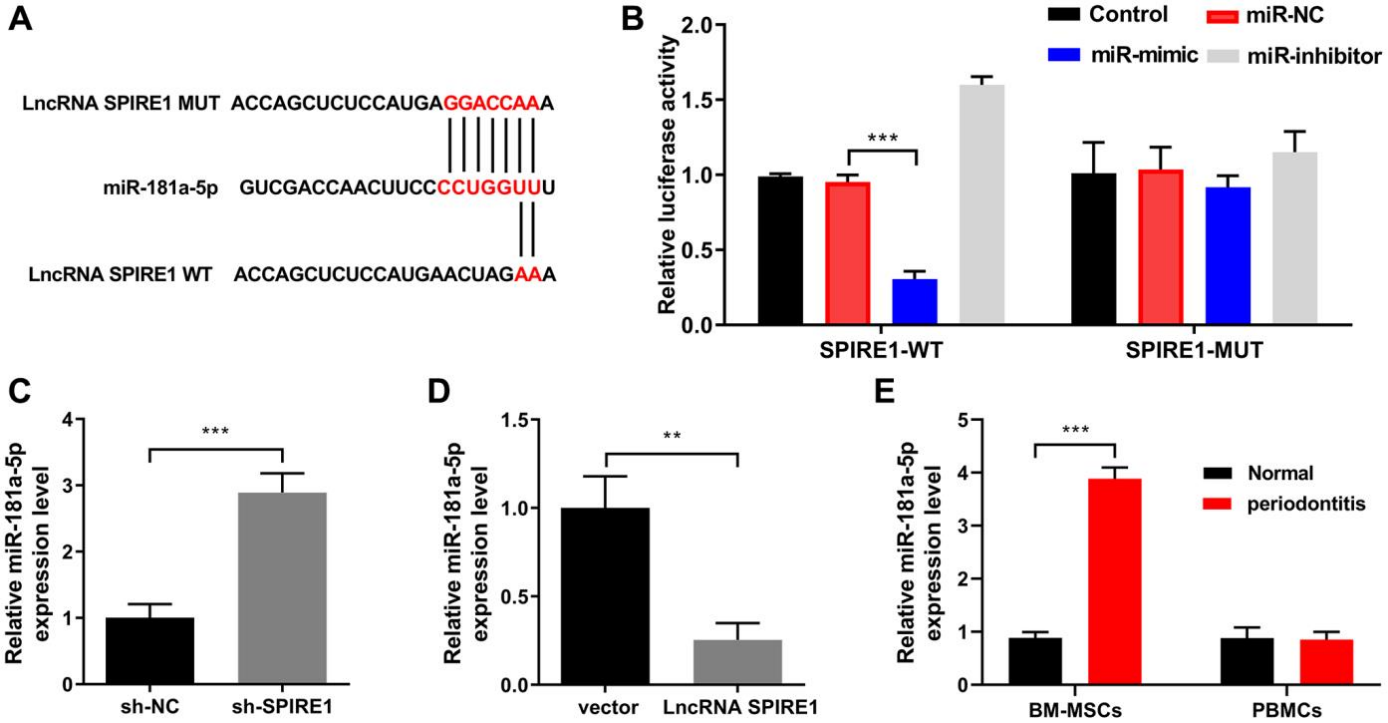
**LncRNA SPIRE1 is a ceRNA that sponges miR-181a-5p in mouse mandibular BM-MSCs.** (**A**) Schematic diagram shows the matching base pairs between miR-181a-5p and the wild type (WT) or mutated (MUT) LncRNA SPIRE1. (**B**) The luciferase activity of the reporter vectors was detected in mandibular BM-MSCs at 48 hours after co-transfection of the plasmid expressing wild type or mutant LncRNA SPIRE1 together with the negative control (NC) mimic or inhibitor, or miR-181a-5p-specific mimic or inhibitor. (**C**, **D**) The relative expression level of miR-181a-5p in mandibular BM-MSCs was determined at 48 hours after the transient transfection of control scramble shRNA or LncRNA SPIRE1-specific shRNA (**C**), or empty vector or LncRNA SPIRE1-expressing plasmid (**D**) by RT-qPCR. (**E**) Mandibular-BM-MSCs (but not blood cells) from periodontitis mice displayed a higher expression level of miR-181a-5p than that from normal controls. The level of miR-181a-5p in BM-MSCs and blood cells of the indicated groups was measured by qPCR. *n* = 3 for each group; ^**^P < 0.01; ^***^P < 0.001, between the indicated groups.

Our *in silico* analysis with the DIANA (http://diana.imis.athena-innovation.gr/DianaTools/index.php) and HMDD (http://www.cuilab.cn/hmdd) tools suggested that miR-181a-5p is a potential miRNA with multiple bases paired to LncRNA SPIRE1 (Figure 3A). To confirm the targeting relationship between these two molecules, we performed luciferase reporter assays in normal mandibular BM-MSCs after transfection of wild type or mutated LncRNA SPIRE1 together with miRNA mimics or inhibitors. As shown in Figure 3B, compared with the control cells with transfection of plasmids only, the cells transfected with wild type LncRNA SPIRE1 and miR-181a-5p mimics demonstrated significantly reduced luciferase activity, while the cells with simultaneous transfection of miR-181a-5p inhibitors had notable increase of luciferase activity. However, these phenomena were abolished in the cells with co-transfection of mutated LncRNA SPIRE1 (Figure 3B), which validated the specificity of sponging miR-181a-5p by LncRNA SPIRE1. Moreover, targeted knockdown (Figure 3C) or overexpression (Figure 3D) of LncRNA SPIRE1 resulted in significantly up-regulated or down-regulated miR-181a-5p expression in normal mandibular BM-MSCs, respectively. In addition, mandibular BM-MSCs from the periodontitis mice displayed obviously increased expression of miR-181a-5p than that from the normal mice (Figure 3E), further consolidating the targeting relationship between LncRNA SPIRE1 and miR-181a-5p.

**Figure 4:**
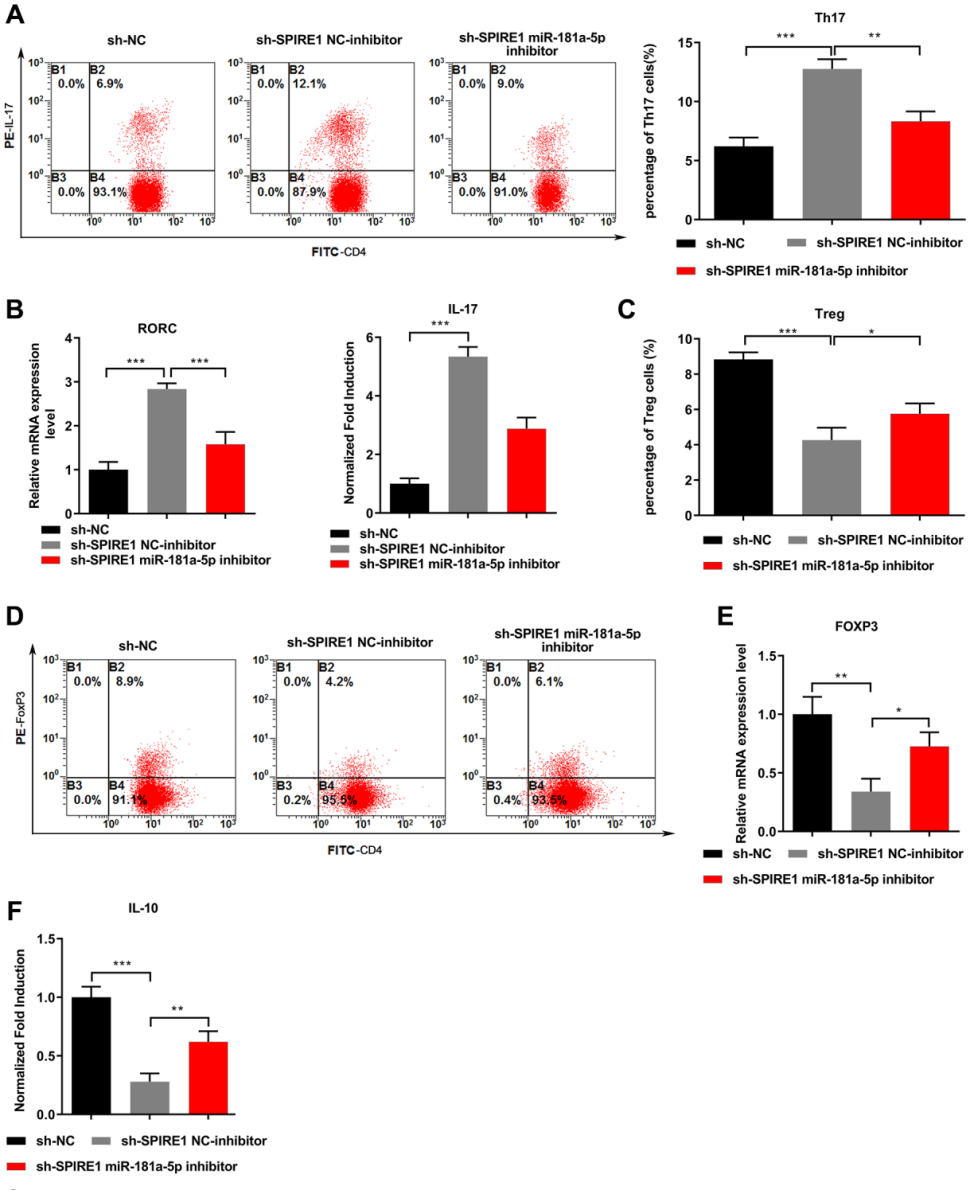
**miR-181a-5p inhibition reversed the influence of LncRNA SPIRE1 knockdown in mouse mandibular BM-MSCs on modulating Th17/Treg balance.** (**A**–**F**) Normal mandibular BM-MSCs were transfected with plasmids expressing negative control shRNA or LncRNA SPIRE1-specific shRNA. At 48 hours after transfection, the LncRNA SPIRE1 knockdown BM-MSCs were further transfected with NC-inhibitor or miR-181a-5p-specific inhibitor. Pre-activated CD4^+^ T cells were co-cultured with the indicated BM-MSCs under the Th17 (**A**–**C**) or Treg (**D**–**F**) polarization condition. Representative flow cytometry profiles of Th17 (**A**) and Treg (**D**) populations, and their percentages are shown. The mRNA levels of RORC (**B**) or FoxP3 (**E**), and the levels of IL-17 (**C**) or IL-10 (**F**) in supernatants were quantitated by qPCR and ELISA, respectively. *n* = 3 for each group; ^*^P < 0.05, ^**^P < 0.01; ^***^P < 0.001, between the indicated groups.

Functional assays in terms of the immune modulation ability of BM-MSCs were also conducted to validate the relationship between LncRNA SPIRE1 and miR-181a-5p. The LncRNA SPIRE1-silenced BM-MSCs were additionally transfected with NC-inhibitor or miR-181a-5p inhibitor, and co-cultured with activated CD4^+^ T cells to evaluate their immune suppressive functions. LncRNA SPIRE1 knockdown alone caused significantly increased percentage of Th17 cells (Figure 4A), upregulated expression of RORC mRNA and release of IL-17 (Figure 4B), while all these trends were greatly diminished upon additional miR-181a-5p inhibition. Similarly, the attenuated immunosuppression ability with a decreased frequency of Treg cells (Figure 4C and 4D), down-regulated FoxP3 expression (Figure 4E), and reduced production of IL-10 (Figure 4F) in BM-MSCs upon LncRNA SPIRE1 knockdown was evidently reversed after further miR-181a-5p inhibition. Therefore, these results together consolidated the notion that miR-181a-5p is a downstream molecule of LncRNA SPIRE1 in controlling the immunomodulation function of BM-MSCs.

### miR-181a-5p targeted PRLR in mandibular BM-MSCs, and PRLR showed down-regulated expression in mandibular BM-MSCs of mice with periodontitis

**Figure 5:**
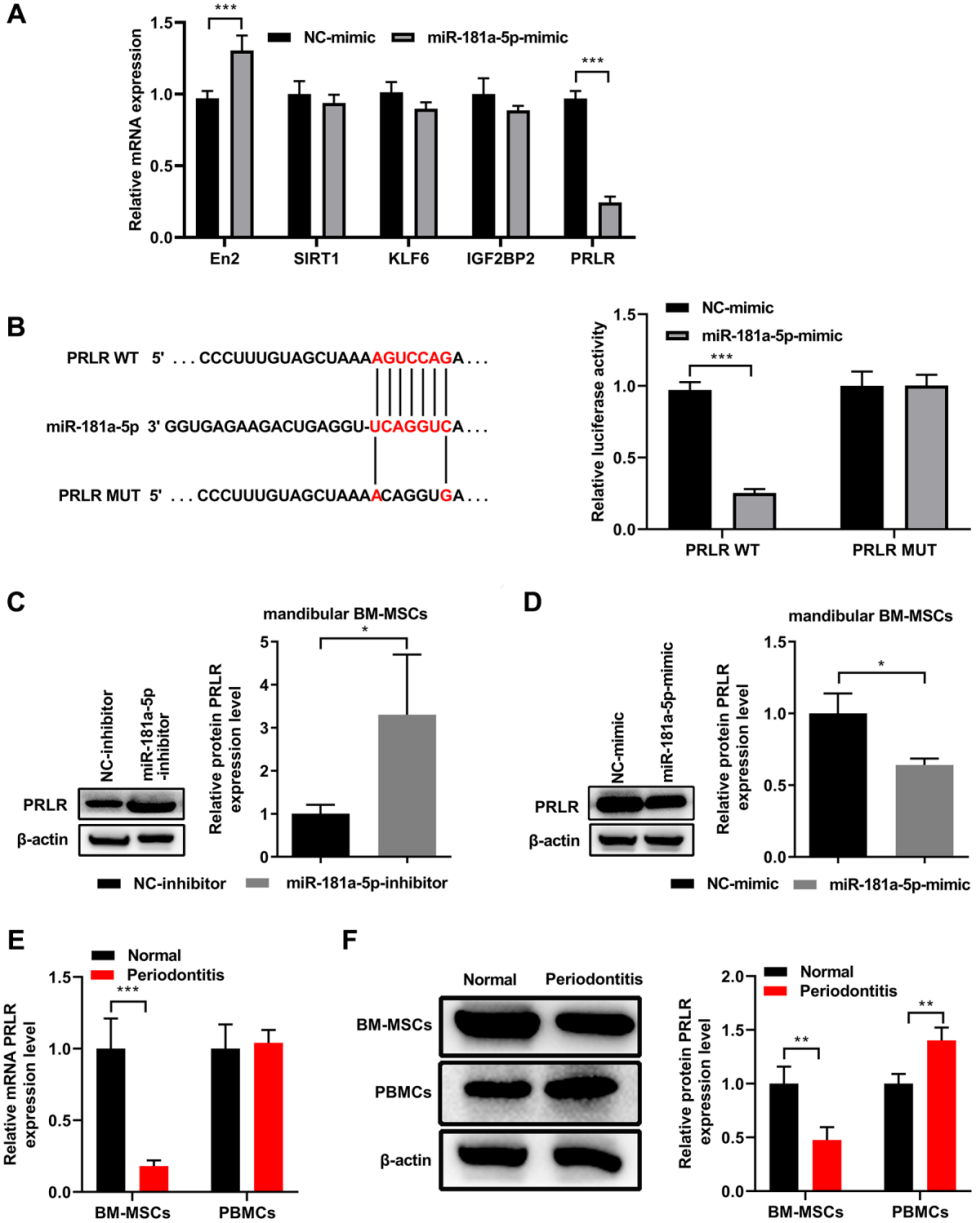
**PRLR is a downstream target of miR-181a-5p in mouse mandibular BM-MSCs.** (**A**) Normal mandibular BM-MSCs were transfected with NC-mimic or miR-181a-5p. At 48 hours after transfection, the mRNA levels of potential miR-181a-5p targeted candidate genes were quantitated by qPCR. (**B**) Schematic diagram shows the matching base pairs between miR-181a-5p and the wild type (WT) or mutated (MUT) 3′UTR of PRLR mRNA. The luciferase activity of the reporter vectors was detected in mandibular BM-MSCs at 48 hours after co-transfection of the plasmid expressing wild type or mutant 3′UTR of PRLR mRNA together with the NC mimic or the miR-181a-5p mimic. (**C**, **D**) Functional inhibition (**C**) or overexpression (**D**) of miR-181a-5p enhanced or reduced PRLR protein expression in mandibular BM-MSCs, respectively. The representative western blot images are shown, and the relative band intensity of PRLR by densitometry was summarized. (**E**, **F**) Mandibular-BM-MSCs (but not blood cells) from mice with periodontitis displayed a lower expression level of PRLR mRNA (**E**) or protein (**F**) than that from normal controls. The mRNA and protein levels were quantitated by qPCR and western blot assays, respectively. *n* = 3 for each group; ^*^P < 0.05, ^***^P < 0.001, between the indicated groups.

To further investigate the molecular mechanisms underlying the immune modulation function of miR-181a-5p, we used open accessible online algorithms to predict the target genes of miR-181a-5p. Subsequent validation with qPCR assays showed that among 5 candidate targets, PRLR is the only one whose mRNA level was significantly downregulated upon transient transfection of miR-181a-5p mimics in normal mandibular BM-MSCs (Figure 5A). The sequence at the 3′UTR of PRLR was highly complementary with the seed sequence of miR-181a-5p (Figure 5B). The dual luciferase assays demonstrated that the luciferase activity was prominently deceased after co-transfection of wild type PRLR 3′UTR and miR-181a-5p mimics, but remained largely unchanged after co-transfection of mutated PRLR 3′UTR and miR-181a-5p mimics in mandibular BM-MSCs, in comparison to the groups with transfections of NC-mimic or PRLR mutated 3′UTR (Figure 5B). These results confirmed the direct binding of miR-181a-5p to the predicted 3′UTR of PRLR mRNA. In addition, compared with the control transfected BM-MSCs, the BM-MSCs with transfection of miR-181a-5p inhibitor (Figure 5C) or miR-181a-5p mimic (Figure 5D) demonstrated markedly upregulated or downregulated expression of PRLR protein, respectively. Furthermore, mandibular BM-MSCs from mice with periodontitis showed significantly down-regulated expressions of PRLR mRNA and protein, as evidenced by qPCR (Figure 5E) and western blot (Figure 5F) assays, respectively. Collectively, these data indicate that RPLE is a direct downstream target of miR-181a-5p in mouse mandibular BM-MSCs, and the expression of RPLE is downregulated upon periodontitis induction.

### The LncRNA SPIRE1/miR-181a-5p/PRLR axis regulated the JAK/STAT3 signaling in mandibular BM-MSCs to modulate the Th17/Treg balance

**Figure 6:**
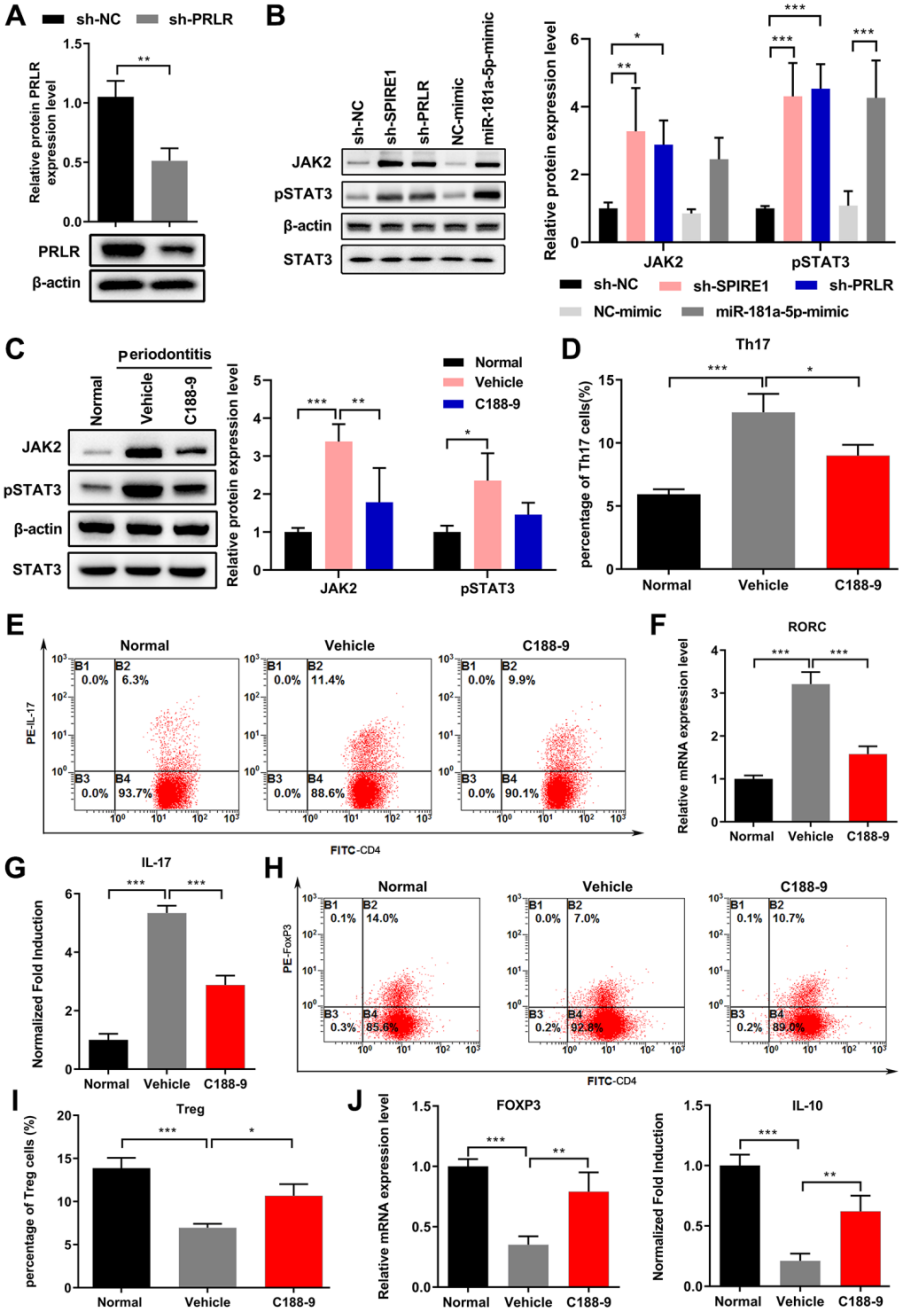
**The LncRNA SPIRE1/miR-181a-5p/PRLR axis regulated the JAK/STAT3 signaling in mouse mandibular BM-MSCs to modulate the Th17/Treg balance.** (**A**) The knockdown efficiency of PRLR-specific shRNA in mandibular BM-MSCs was confirmed by qPCR analysis of BM-MSCs transfected with plasmid expressing the indicated shRNA. (**B**) Knockdown of LncRNA SPIRE1 or PRLR, or transfection of miR-181a-5p mimics activated the JAK/STAT3 pathway, as evidenced by increased expression of JAK2 and phosphorylated STAT3 (pSTAT3). The protein levels of JAK2 and pSTAT3 in mandibular BM-MSCs after the indicated transfections were quantitated by western blot assays. (**C**) Mandibular BM-MSCs from periodontitis mice had more activated JAK/STAT3 signaling than that from normal controls, which was inhibited by the STAT3 inhibitor C188-9. The protein levels of JAK2 and pSTAT3 were quantitated as in (**B**). (**D**–**J**) Mandibular BM-MSCs from periodontitis mice were treated with vehicle or STAT3 inhibitor C188-9, while mandibular BM-MSCs from normal control mice were used as controls. After co-culturing of these BM-MSCs with pre-activated CD4^+^ T cells, the percentages of CD4^+^IL-17^+^ Th17 cells (**D**, **E**), RORC mRNA levels (**F**), soluble IL-17 level in culture medium (**G**), as well as the percentages of CD4^+^FoxP3^+^ Tregs (**H**, **I**), FoxP3 mRNA levels and soluble IL-10 levels in culture medium (**J**) were measured. *n* = 3 for each group; ^*^P < 0.05, ^**^P < 0.01; ^***^P < 0.001, between the indicated groups.

Since the JAK/STAT3 signaling was reported to regulate the inflammatory cascade and influence the adaptive immunity by modulating Th17/Treg cell differentiation [33], we investigated whether the LncRNA SPIRE1/miR-181a-5p/PRLR axis were associated with this signaling. First, we validated the efficacy of shRNA-mediated knockdown of PRLR in normal mandibular BM-MSCs (Figure 6A), and examined the impact of LncRNA SPIRE1 knockdown or PRLR knockdown, as well as the transient transfection of miR-181a-5p mimic, on regulation of the JAK/STAT3 signaling pathway. As shown in Figure 6B, periodontitis-associated changes in gene expressions, including downregulated expressions of LncRNA SPIRE1 or PRLR, and increased miR-181a-5p expression, resulted in significantly activated JAK/STAT3 signaling in normal BM-MSCs, in comparison to the transfected control cells. In addition, mandibular BM-MSCs from periodontitis mice also showed markedly more activated JAK/STAT3 signaling, as evidenced by higher levels of JAK2 and phosphorylated STAT3 proteins (Figure 6C). However, the treatment with C188-9, a synthetic small molecule that specifically inhibits the STAT3 pathway [35], showed obvious suppression of this pathway in BM-MSCs from periodontitis mice (Figure 6C). To validate the contribution of the activated JAK/STAT3 signaling on the impaired immune modulation ability of BM-MSCs from periodontitis mice, we additionally treated these BM-MSCs with C188-9 before and during their co-culturing with the CD4^+^ T cells. Inactivation of the STAT3 signaling remarkably restored the ability of periodontitis mandibular-derived BM-MSCs in immune suppression, as evidenced by significant reductions in the ratio of Th17 population (Figure 6D and 6E), decreased expression of RORC mRNA (Figure 6F), reduced production of IL-17 (Figure 6G), as well as increased ratio of Treg cells (Figure 6H and Figure 6I), increased FoxP3 mRNA expression and increase secretion of IL-10 (Figure 6J), when compared to the group with vehicle treatment.

**Figure 7:**
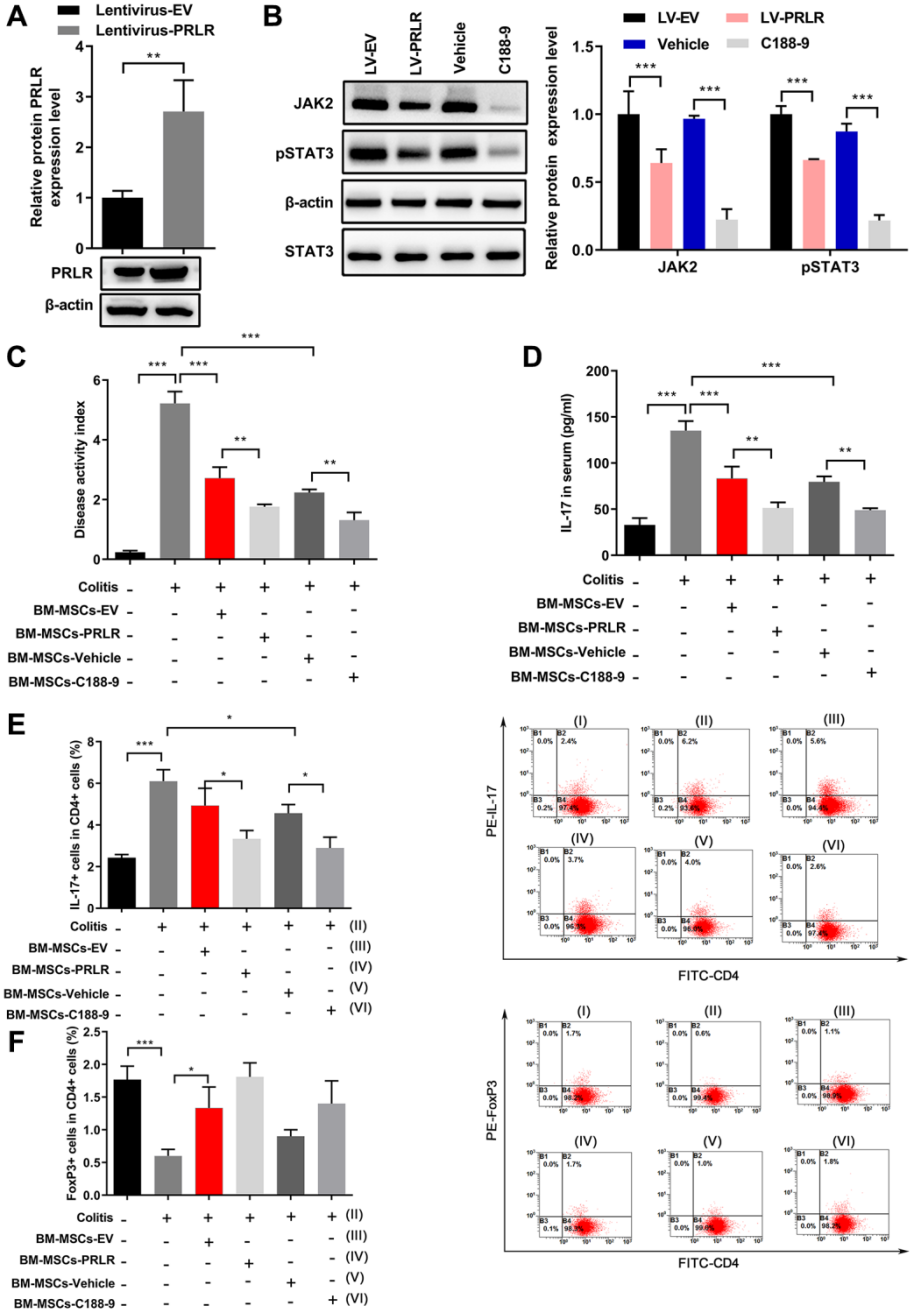
**C188-9 treatment enhanced the effect of PRLR on regulation of Th17/Treg balance.** (**A**) Confirmation of overexpression of PRLR in BM-MSCs using western blotting. (**B**) The protein levels of JAK2 and pSTAT3 in mandibular BM-MSCs were quantitated by western blot assays after overexpression of PRLR or treatment with C188-9. (**C**–**F**) Mandibular BM-MSCs from periodontitis mice were infected with PRLR-expressing lentivirus or empty vector virus, or treated with STAT3 inhibitor C188-9 or vehicle. Acute colitis in mice was induced by administering 3% (w/v) dextran sulfate sodium (DSS) through drinking water, and mice were fed *ad libitum* for 10 days. Control BM-MSCs or PRLR-overexpressing BM-MSCs, or C118-9-pretreated BM-MSCs (0.2 × 10^6^ cells) were infused into colitis mice (*n* = 5 mice per group) intravenously at day 3 after feeding DSS water. Serum and blood samples from all mice were harvested at day 10 after the DSS water was provided. (**C**) Disease activity index was measured at 7 days after infusion of BM-MSCs with overexpression of PRLR or with C188-9 treatment. (**D**) Soluble IL-17 level in mouse serum was measured by ELISA. (**E**, **F**) The percentages of CD4^+^IL-17^+^ (**E**), and the percentages of CD4^+^FoxP3^+^ Tregs (**F**) among blood CD4^+^ T cells from mice of the indicated groups were measured. *n* = 5 for each group; ^*^P < 0.05, ^**^P < 0.01; ^***^P < 0.001, between the indicated groups.

In addition, PRLR was also overexpressed to further confirm its role in the periodontitis development (Figure 7A). PRLR overexpression significantly decreased the JAK/STAT3 signaling, while C188-9 treatment obviously enhanced these changes (Figure 7B). Overexpression of PRLR significantly decreased the inflammatory injury and serum IL-17 levels after BM-MSCs infusion under inflammatory environment in an acute colitis mouse model, and C188-9 treatment of BM-MSCs also resulted in similar changes (Figure 7C and 7D). Further analyses presented that PRLR overexpression in BM-MSCs markedly decreased the percentages of IL-17^+^ cells but decreased the percentages of FoxP3^+^ cells among mouse blood CD4^+^ cells, while C188-9 treatment of BM-MSCs led to similar changes, in comparison to the respective control treatment (Figure 7E and 7F). Therefore, the LncRNA SPIRE1/miR-181a-5p/PRLR axis regulated the JAK/STAT3 signaling in mandibular BM-MSCs to modulate the Th17/Treg balance.

### Targeted regulation of the LncRNA SPIRE1/miR-181a-5p/PRLR axis in mandibular BM-MSCs enhanced the immunomodulation and healing in mice with periodontitis

**Figure 8:**
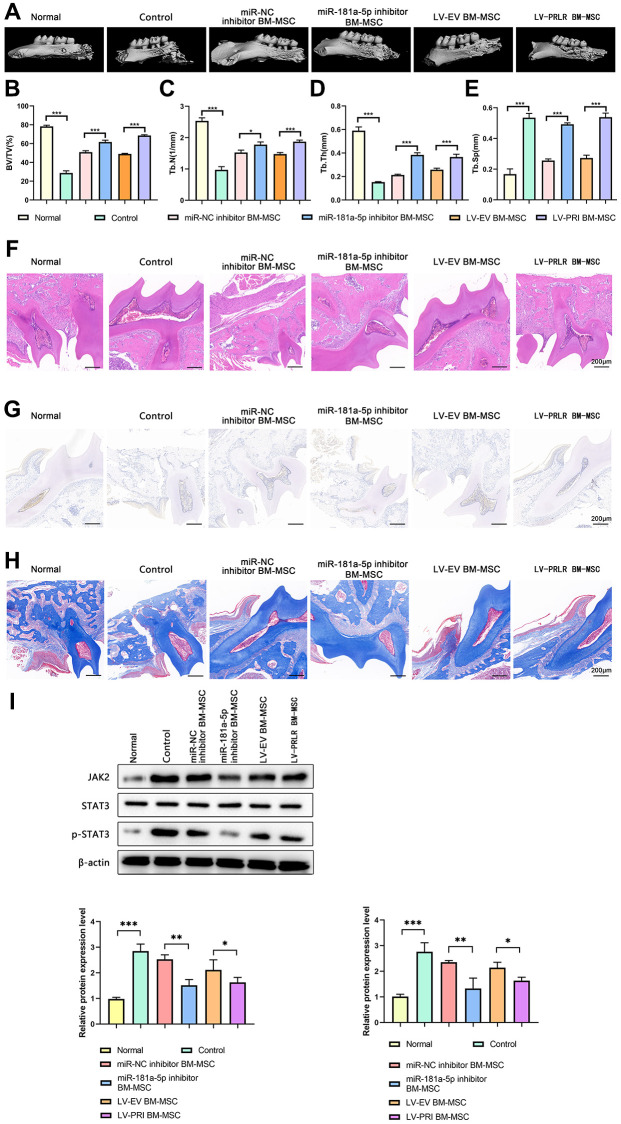
**Targeted manipulation of the LncRNA SPIRE1/miR-181a-5p/PRLR pathway in BM-MSCs enhanced their immunomodulation in mice with periodontitis.** (**A**–**I**) After disease induction, mice were left un-treated, or infused with control mandibular BM-MSCs, or the mandibular BM-MSCs transfected with miR-181a-5p inhibitor, or the mandibular BM-MSC infected with control lentivirus or PRLR-expressing lentivirus as indicated. The up-regulated expression of PRLR after infection of PRLR-expressing lentivirus was confirmed by western blot assays. (**A**) Micro-CT images show the osteogenesis around the periodontal tissues. (**B**–**E**) The BV/TV (%) (**B**), Tb.N1 (**C**), Tb.Th (**D**), and Tb.Sp (**E**) in mice of the indicated groups were summarized. (**F**–**H**) Histological analyses of the periodontal tissues by H&E staining (**F**), TRAP staining (**G**), and Masson staining (**H**) were conducted. The representative images of each group are shown. (**I**) The protein levels of JAK2 and pSTAT3 in periodontal tissues of the indicated groups were quantitated by western blot assays. *n* = 5 mice for each group; ^*^P < 0.05, ^**^P < 0.01; ^***^P < 0.001, between the indicated groups.

To further evaluate the *in vivo* benefits of targeted regulation of the LncRNA SPIRE1/miR-181a-5p/PRLR axis in immune modulation, we investigated the immunosuppressive properties of mandibular BM-MSCs after gene manipulations in LPS-induced experimental periodontitis mouse model. These mice with periodontitis were divided to control groups and the groups with infusion of mandibular BM-MSCs after targeted inhibition of miR-181a-5p or overexpression of PRLR. An overall assessment of the therapeutic efficacy was based on the image data which reflected the osteogenesis of periodontal bones (Figure 8A). Compared with the un-treated group, the groups treated with control BM-MSCs showed significantly reduced bone loss rates and considerably increased bone regeneration. Notably, the groups with infusion of miR-181a-5p inactivated BM-MSCs or PRLR-overexpressing BM-MSCs demonstrated more reduction in bone loss and more increase in bone regeneration (Figure 8B–8E), suggesting the enhanced immunomodulatory properties of the genetically modified BM-MSCs.

Further histological examination by H&E staining (Figure 8F) and TRAP staining (Figure 8G) showed that the un-treated group displayed severe colonic transmural inflammation with increased wall thickness, infiltration of inflammatory cells, degeneration of crypt architecture, and loss of osteoblast cells. Notably, the treated groups with BM-MSCs infusion had significantly reduced number of osteoclasts than the un-treated group, while the group with infusion of the miR-181a-5p or PRLR expression-altered BM-MSCs showed the lowest number of osteoclasts in mouse periodontal tissues. Consistently, periodontal regeneration observed by Masson’s trichrome staining also showed that these groups had the best osteogenic differentiation of bone progenitor cells (Figure 8H). Moreover, the protein quantitation by western blot analysis showed that the periodontal tissues from the mice infused with the miR-181a-5p or PRLR expression-altered BM-MSCs demonstrated significantly reduced activity of JAK/STAT3 signaling (Figure 8I), strengthening the notion that inhibition of the JAK/STAT3 signaling pathway in BM-MSCs enhanced their immunomodulation ability.

## DISCUSSION

The balance between Th17 and Treg cells plays critical roles in maintaining immune tolerance, and the break of this balance has been implicated in the pathophysiology of the development of periodontitis [8, 11, 33]. Due to the potent immunomodulatory properties which cover the modulating ability of the Th17/Treg balance, BM-MSCs hold great promise in clinical applications for treating immune-dysfunction-related diseases like periodontitis. However, how to boost the immunosuppressive properties of normal BM-MSCs or to rescue the defective immunomodulation ability of BM-MSCs under the periodontitis settings remains poorly understood. In this study, we identified LncRNA SPIRE1 as an ncRNA that was significantly down-regulated in mandibular BM-MSCs from periodontitis mice, and knockdown of LncRNA SPIRE1 from normal mandibular BM-MSCs recapitulated their counterparts in periodontitis mice in term of inducing Th17/Treg imbalance. Our further investigation showed that LncRNA SPIRE1 is a ceRNA that sponges miR-181-5p, which targeted PRLR to regulate the immunosuppressive functions of BM-MSCs through the JAK/STAT3 pathway. In addition, we demonstrated the feasibility and efficacy of using genetically modified mandibular BM-MSCs for treating periodontitis in a mouse model, which provides valuable insight in employing mandibular BM-MSCs in future clinical applications.

Although bacterial infection, as well as genetic and environmental parameters, have been acknowledged to contribute to the initiation and development of periodontitis, the critical roles of lncRNAs in the pathogenesis of periodontitis have been demonstrated by recent studies [29, 36]. Through modulating chromatin configuration, epigenetic control of gene expression, gene transcription and translation, as well as RNA stability, lncRNAs play significant roles under both physiologic and pathological conditions [29, 36]. Accumulating investigations reported the aberrant expression of lncRNAs in tissues samples from patients with periodontitis. In this study, we found that the mandibular BM-MSCs derived from the mice with induced periodontitis showed a higher expression level of LncRNA SPIRE1 than that from healthy mice. However, the peripheral blood samples had a comparable level of LncRNA SPIRE1. Since blood is comprised of mainly hematopoietic cells with multiple subpopulations, our results could not tell the possible altered levels of LncRNA SPIRE1 in specific immune cell types. A consistent pattern on altered lncRNA expression between blood samples and dental tissues had been noticed in some reports. For example, the expression of LncRNA NKILA was higher in both the gingival tissue samples and blood samples of patients compared with controls [37]. These observations suggest that lncRNAs might possess some tissue-specific functions, while the role of LncRNA SPIRE1 in the development of periodontitis might be more reflected in periodontal MSCs. Indeed, our further investigation showed that knockdown of LncRNA SPIRE1 impaired the immunosuppressive ability of BM-MSCs *in vitro*. Nevertheless, the functions of LncRNA SPIRE1 in other periodontal tissues such as periodontal ligament cells, gingival tissue, as well as the association between LncRNA SPIRE1 expression and the environmental parameters, need to be further studied to warrant a more comprehensive understanding on the roles of LncRNA SPIRE1 in periodontitis.

MicroRNAs have been widely reported to play a critical role in the differentiation of MSCs, including both positive and negative regulation [38]. The miR-181 family, a group of highly conserved miRNAs of growing biomedical relevance, regulates many relevant biological processes such as cell proliferation, apoptosis, autophagy, mitochondrial function, and immune response [39]. miR-181a-5p was reported to function as an oncogene in cancers like renal cell carcinoma [40] and to regulate inflammatory response in sepsis [41] and vascular inflammation [42]. It’s role in MSC immunomodulation has not been intensively studied so far. Here, we identified miR-185-5p as a sponging target of LncRNA SPIRE1, and notably, as a pathogenic factor in periodontitis development, as its inhibition could significantly restore the impaired immunomodulation ability of mandibular BM-MSCs. In murine macrophage cell line RAW264.7, LPS stimulation upregulated miR-181a-5p expression, and miR-181a-5p inhibitor significantly suppressed LPS-enhanced inflammatory cytokines production and NF-κB pathway activation [41]. In another report, Su et al. found that miR-185-5p was involved in reducing endothelium inflammation through blockade of NF-κB signaling pathway to prevent endothelial cell activation [42]. The distinct roles of miR-181a-5p in macrophages and endothelial cells suggest that the function of miR-181a-5p in regulating inflammation is more like cellular context-dependent. Recently, it was revealed that overexpressing miR-181a-5p considerably reduced the cell growth and increased apoptosis of human BM-MSCs through regulating the Sirt1/PI3K/AKT signaling pathway [43]. Therefore, whether the attenuated Th17/Treg imbalance by miR-181a-5p inhibition in mandibular BM-MSC was related to enhanced cell proliferation and reduced apoptosis remains to be further elucidated.

Multiple targets have been identified to regulate the inflammatory responses in different tissues and cells, such as SIRT1 in murine myeloid cells [41] and HMGB1 in rat adrenal gland cells [44]. In this study, PRLR was confirmed as one downstream target of miR-181a-5p in mandibular BM-MSCs, and its overexpression was found to improve the immunomodulatory properties of BM-MSCs. PRLR is a type 1 cytokine receptor and may contribute to the pathogenesis of multiple cancers. For example, PRLR is highly expressed in a subset of human breast cancer and prostate cancer, which makes it a potential target for cancer treatment [45]. Prolactin (PRL)-dependent signaling occurs as the result of ligand-induced dimerization of the prolactin receptor, their interaction can modulate the endocrine and autocrine effects of prolactin in normal tissue and cancer [46, 47]. However, whether and how PRLR is involved in MSC immunomodulation is not known. PRL has been associated with the modulation of a variety of actions in the immune response and inflammatory processes in several physiologic and pathologic conditions [48]. For example, in rheumatoid arthritis and psoriatic arthritis, PRL can be locally produced by macrophages, T cells and synovial fibroblasts while PRLR is expressed in synovial macrophages, lymphocytes, and fibroblasts [49]. Data showed that PRL enhances the expression of several genes encoding for pro-inflammatory cytokines (IL-6, IL-8, IL-12β, TNF) and chemokines (i.e., CXCL3, 5, 6, and 11) in macrophages [49]. On the contrary, PRLR appeared to be an anti-inflammatory factor in our experimental settings, since a lower expression level of PRLR was observed in mandibular BM-MSCs derived from periodontitis mice than that from normal mice. Therefore, these results suggest that the PRLR-PRL interaction might enhance or inhibit pro-inflammatory cytokine production in a cell-specific manner. Furthermore, since PRL is involved in regulation of adult stem/progenitor cells homeostasis [50] and osteogenic differentiation of adipose tissue-derived MSCs [51], the contribution of PRL in the novel axis of LncRNA SPIRE1/miR-181a-5p/PRLR in immunoregulation remains to be explored.

The JAK/STAT signaling pathway has been implicated in the pathogenesis of inflammatory and autoimmune diseases including rheumatoid arthritis, psoriasis, and inflammatory bowel disease [52]. Prolactin-induced production of cytokines was reported to involve the activation of JAK/STAT pathway in murine peritoneal macrophages [53]. However, we found that knockdown of PRLR led to the activation of the JAK2/STAT3 signaling in BM-MSCs, suggesting that PRLR might utilize other mechanisms to regulate the JAK/STAT signaling rather than that initiated by the RPL/RPLR interaction in MSCs. It is also possible that the PRLR-PRL interaction might enhance or inhibit inflammation through different mechanisms in different cell types. The crucial role of the STAT3 pathway in orchestrating Th17/Treg differentiation has been validated in many studies, and it was reported that through coordinating the expression of inflammatory factors, the STAT3 signaling controls the differentiation of naïve CD4 T cell families (Th1, Th2, Th17, and Treg) [54]. Consistent with previous findings, we found that a higher level of STAT3 signaling in BM-MSCs from periodontitis mice can induce more IL-17 production and higher expression levels of RORC in CD4 T cells. Targeted blocking of the STAT signaling by C188-9 was able to abrogate the Th17/Treg balance under the periodontitis settings. Therefore, the use of STAT3 inhibitors can interrupt the pro-inflammatory process and have an antagonistic effect on periodontitis-associated inflammation, as previously reported [33]. Recently, it was reported that STAT3 inhibition by the inhibitor WP1066 resulted in increased expression levels of miR-181a-5p in glioblastoma multiforme cell lines [55]. Whether a similar feedback loop or counter action/balance between the expression of miR-181a-5p and JAK/STAT3 signaling exists in mandibular BM-MSCs is intriguing for further exploration.

In summary, through the *in vivo* mouse model of *P. gingivalis* LPS-induced periodontitis and *in vitro* co-culture system of mandibular BM-MSCs and pre-activated splenic CD4 T cells, we revealed a novel axis of LncRNA SPIRE1/miR-181a-5p/PRLR in regulating the immunomodulatory properties of mandibular BM-MSCs. Our results indicate that the JAK/STAT3 pathway is involved in the immunoregulation of BM-MSCs, and provide critical insight into the development of novel targeted therapies against periodontitis and perhaps also other inflammation/immune disorder-related diseases.

## Supplementary Materials

Supplementary Figure 1

Supplementary Table 1
