# Supplementary material for: A novel LncRNA SPIRE1/miR-181a-5p/PRLR axis in mandibular bone marrow–derived mesenchymal stem cells regulates the Th17/Treg immune balance through the JAK/STAT3 pathway in periodontitis
Source: Aging (Albany NY). 2023 Jul 24;15(14):7124–45. doi: 10.18632/aging.204895 (PMC10415575; doi:10.18632/aging.204895)
Supplement: Supplementary Figure 1 [file aging-15-204895-s001.pdf]

SUPPLEMENTARY FIGURE

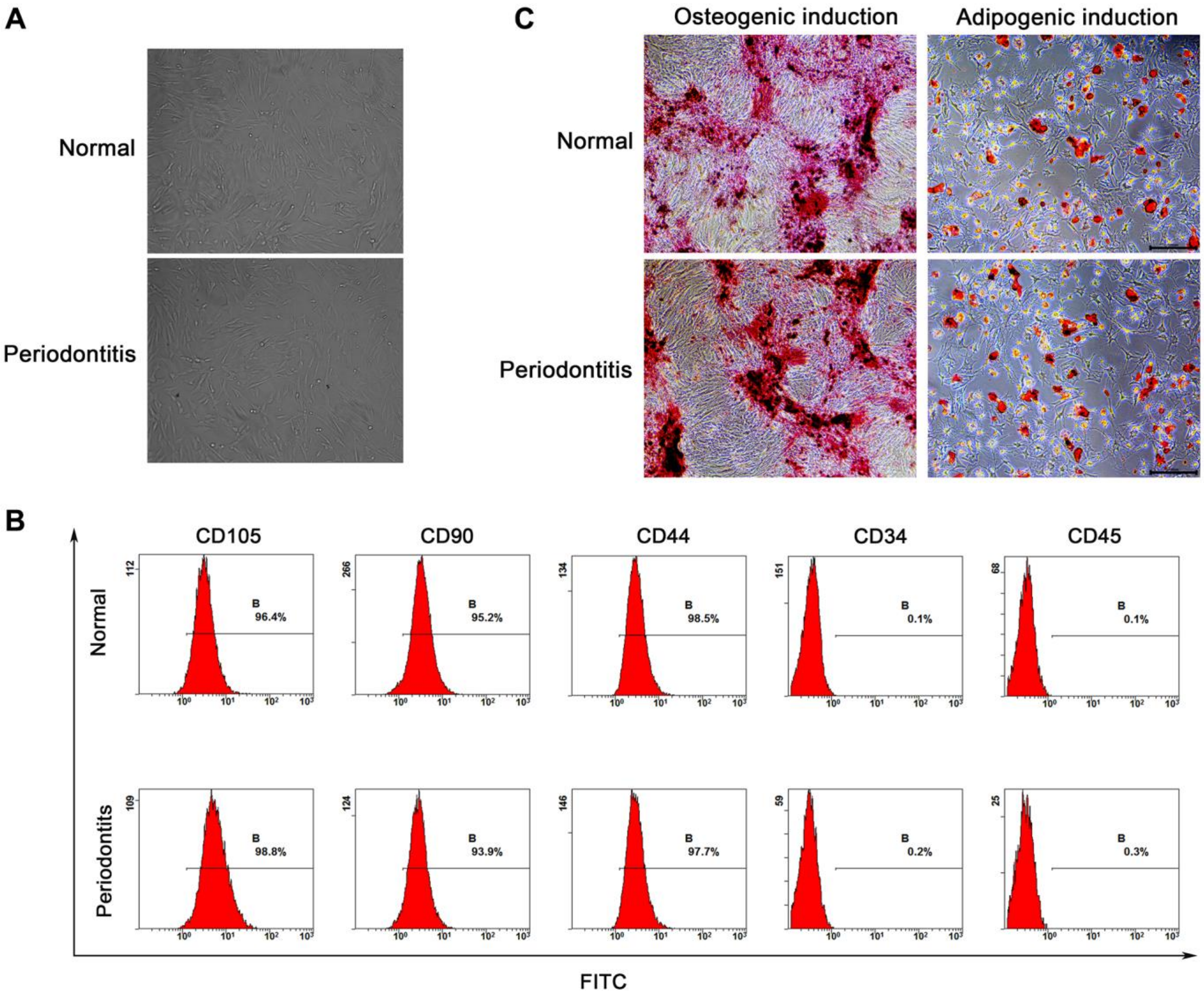

Supplementary Figure 1. Characterization of mandibular BM-MSCs from periodontitis healthy normal mice or mice induced with periodontitis. (**A**) Cell morphology of mandibular bone marrow–derived MSCs at Day 14 after culturing. (**B**) Flow cytometric analysis of MSC markers and hematopoiesis markers in normal/periodontitis mandibular BM-MSCs. These BM-MSCs were $CD105^{+}CD90^{+}CD44^{+}CD34^{-}CD45^{-}$. All Abs were PE-conjugated and were bought from BioLegend. (**C**) Multilineage differentiation capacity of established mandibular BM-MSCs. Osteogenic differentiation was confirmed by the formation of mineralized matrix in both normal/periodontitis mandibular BM-MSCs after osteogenic induction (40× magnification). Adipogenic differentiation was confirmed by oil red O staining. Lipid vacuoles cells were found in the images after adipogenic induction (200×).
