# Supplementary material for: A novel LncRNA SPIRE1/miR-181a-5p/PRLR axis in mandibular bone marrow–derived mesenchymal stem cells regulates the Th17/Treg immune balance through the JAK/STAT3 pathway in periodontitis
Source: Aging (Albany NY). 2023 Jul 24;15(14):7124–45. doi: 10.18632/aging.204895 (PMC10415575; doi:10.18632/aging.204895)
Supplement: Supplementary Table 1 [file aging-15-204895-s002.pdf]

SUPPLEMENTARY TABLE

Supplementary Table 1. Primers for real-time PCR in this study.

Gene	Forward primers (5′→3′)	Reverse primers (5′→3′)
RORC	GAGAAGGACAGGGAGCCAAG	GCGGAAGAAGCCCTTGCAC
lncRNA SPIRE1	TTTCCTCAACTGGAGCTGGT	TCGTGCTTCCTGTATTGCAGA
FOXP3	CCCACACTGCCCCTAGTCAT	TCCACCGTTGAGAGCTGGT
miR-181a-5p	AACAUUCAACGCUGUCGGUGAGU	Universal PCR Reverse Primer (#B532451; Sangon Biotech Co., Ltd.)
PRLR	CACTCCTTCTCCCTCTTTCTGG	ATAGGAGAGTTCTTTAGTTTTGCCA
IGF2BP2	AGTGGAATTGCATGGGAAAATCA	CAACGGCGGTTTCTGTGTC
KLF6	GGCAACAGACCTGCCTAGAG	CTCCCGAGCCAGAATGATTTT
SIRT1	TAGCCTTGTCAGATAAGGAAGGA	ACAGCTTCACAGTCAACTTTGT
EN2	CCGGCGTGGGTCTACTGTA	CCTCTTTGTTCGGGTTCTTCTT
GAPDH	AACTTTGGCATTGTGGAAGG	ATTGGGGGTAGGAACA
U6	CTCGCTTCGGCAGCACA	ACGCTTCACGAATTTGCGT
